# Supplementary material for: Genome-wide Cas9-mediated screening of essential non-coding regulatory elements via libraries of paired single-guide RNAs
Source: Nat Biomed Eng. 2024 May 22;8(7):890–908. doi: 10.1038/s41551-024-01204-8 (PMC11310080; doi:10.1038/s41551-024-01204-8)

nature biomedical engineering

Supplementary information https://doi.org/10.1038/s41551-024-01204-8

Genome-wide Cas9-mediated screening of essential non-coding regulatory elements via libraries of paired single-guide RNAs

In the format provided by the authors and unedited

Contents

Supplementary figures
Supplementary Fig. 1 | UCE dual-CRISPR screening in K562 cells.
Supplementary Fig. 2 | Cell viability using single guide-direct CRISPR editing system.
Supplementary Fig. 3 | Epigenetic patterns of validated essential K562 UCEs.
Supplementary Fig. 4 | Essential UCEs and NCREs shared between K562 and 293T cells.
Supplementary Fig. 5 | Cell cycle and apoptosis analysis of essential UCE/NCRE-KO clones.
Supplementary Fig. 6 | Luciferase assays to determine the effects of SNPs on the silencer activity of QKI_Jo.
Supplementary Fig. 7 | Identifying essential enhancer clusters.
Supplementary Fig. 8 | Single-cell dual-CRISPR screening.
Supplementary Fig. 9 | Removing UCE PAX6 affecting cardiac development.

Supplementary tables (additional file)
Supplementary Table 1 | Essential ultra-conserved elements in K562 cells from pooled dual-CRISPR screening.
Supplementary Table 2 | Essential ultra-conserved elements in 293T cells from pooled dual-CRISPR screening.
Supplementary Table 3 | Imatinib-resistance ultra-conserved elements in K562 cells from pooled dual-CRISPR screening.
Supplementary Table 4 | Essential putative K562 enhancers identified by pooled dual-CRISPR screening.
Supplementary Table 5 | Essential K562 enhancer clusters identified by pooled dual-CRISPR screening.
Supplementary Table 6 | Statistics of captured single cells from sc-dual-CRISPR screening.
Supplementary Table 7 | Significant NCRE-gene pairs identified from sc-dual-CRISPR screening.
Supplementary Table 8 | Primers list of dual-CRISPR system, PCR, luciferase assay and qPCR.
***Note:** All Supplementary Tables are provided as one merged .xlsx file.*

Supplementary video captions
Removing PAX6_Ta causes defects in cardiomyocyte differentiation. (Scale bars: 275 µm).
Supplementary Video 1 | NKX2-5eGFP/w hESC WT GFP channel.
Supplementary Video 2 | NKX2-5eGFP/w hESC WT brightfield.
Supplementary Video 3 | NKX2-5eGFP/w hESC PAX6_Ta KO GFP channel.
Supplementary Video 4 | NKX2-5eGFP/w hESC PAX6_Ta KO brightfield.
***Note:** All Supplementary Videos are attached as separated .mp4 files.*

Additional Information
Source Data for Supplementary Fig.1a | Unprocessed Western blot of Cas9-FLAG and Actin.

Supplementary figures

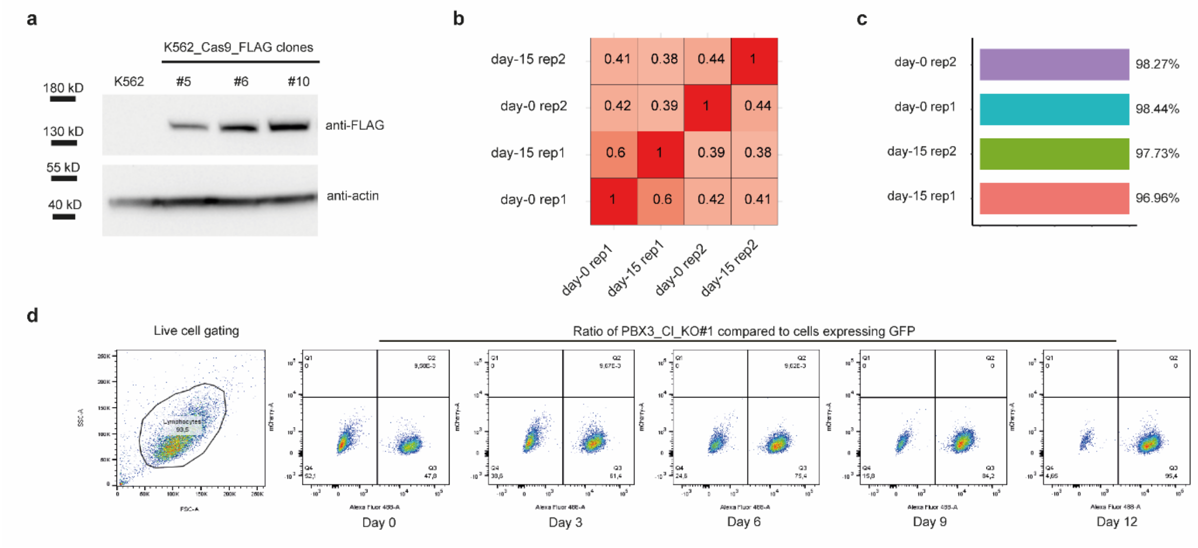

Supplementary Fig. 1 | UCE dual-CRISPR screening in K562 cells. a, Western blot to validate the Cas9-expressing K562 cells. The expression of Cas9-FLAG proteins in different clones was probed with the anti-FLAG antibody. Actin was used as the loading control. K562_Cas9_FLAG clone #10 was used for subsequent screenings. **b,** Spearman correlation between biological replicates of the dual-CIRPSR libraries (day-0: initial screening cell population; day-15: cells grew for another 15 days). **c,** Percentage of the recovered target UCEs and validated enhancers from different samples during the dual-CRISPR screenings in K562 cells. **d,** FACS gating examples of cell proliferation assays.

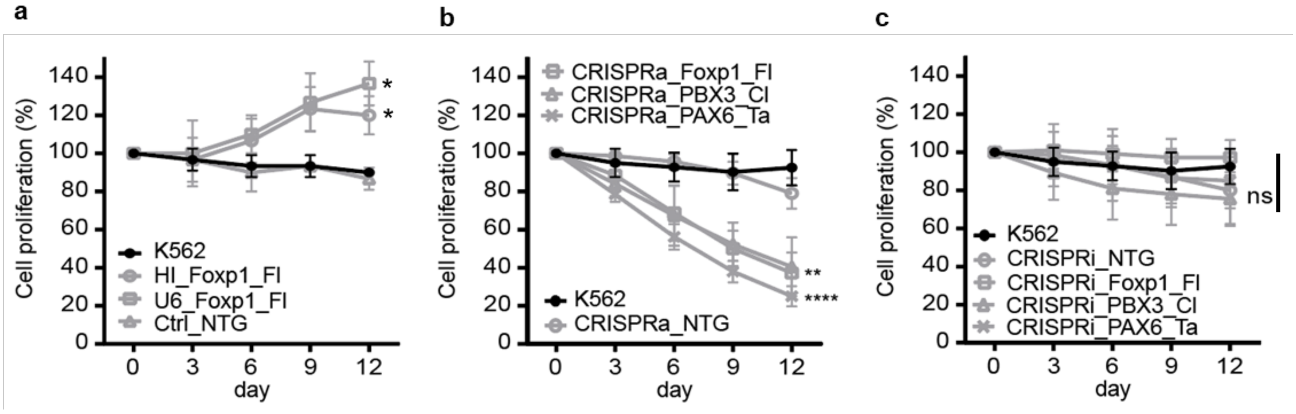

Supplementary Fig. 2 | Cell viability using single guide-direct CRISPR editing system. a, Cell growth effect of CRISPR/Cas9 editing using individual sgRNAs (U6 and H1) from dual-CRISPR guide pair targeting Foxp1_Fl, and control non-targeting guide (NTG). **b,** Cell growth effect of dCas9-VP64 activation system (CRISPRa) using 3 individual sgRNAs to tile Foxp1_Fl, PBX3_Cl and PAX6_Ta regions, and NTG. **c,** Cell growth effect of dCas9-KRAB repression system (CRISPRi) using 3 individual sgRNAs to tile Foxp1_Fl, PBX3_Cl and PAX6_Ta regions, and NTG (c). Cell proliferation assay was performed by mixing the KO cell lines with cells expressing GFP at a 1:1 ratio. The changes in GFP percentage were monitored at indicated time points by FACS. Cells with CRISPR guide RNA targeting non-human sequences served as negative controls (NTG). The y axis represents the relative ratio of the GFP negative cells to the positive cells. The ratio of cells in the initial mixture was set as 100% (n=3 biological independent samples; values are shown as the mean ± s.d.; *P < 0.05, **P < 0.01, ****P < 0.0001, ns: not significant, calculated using two-way ANOVA).

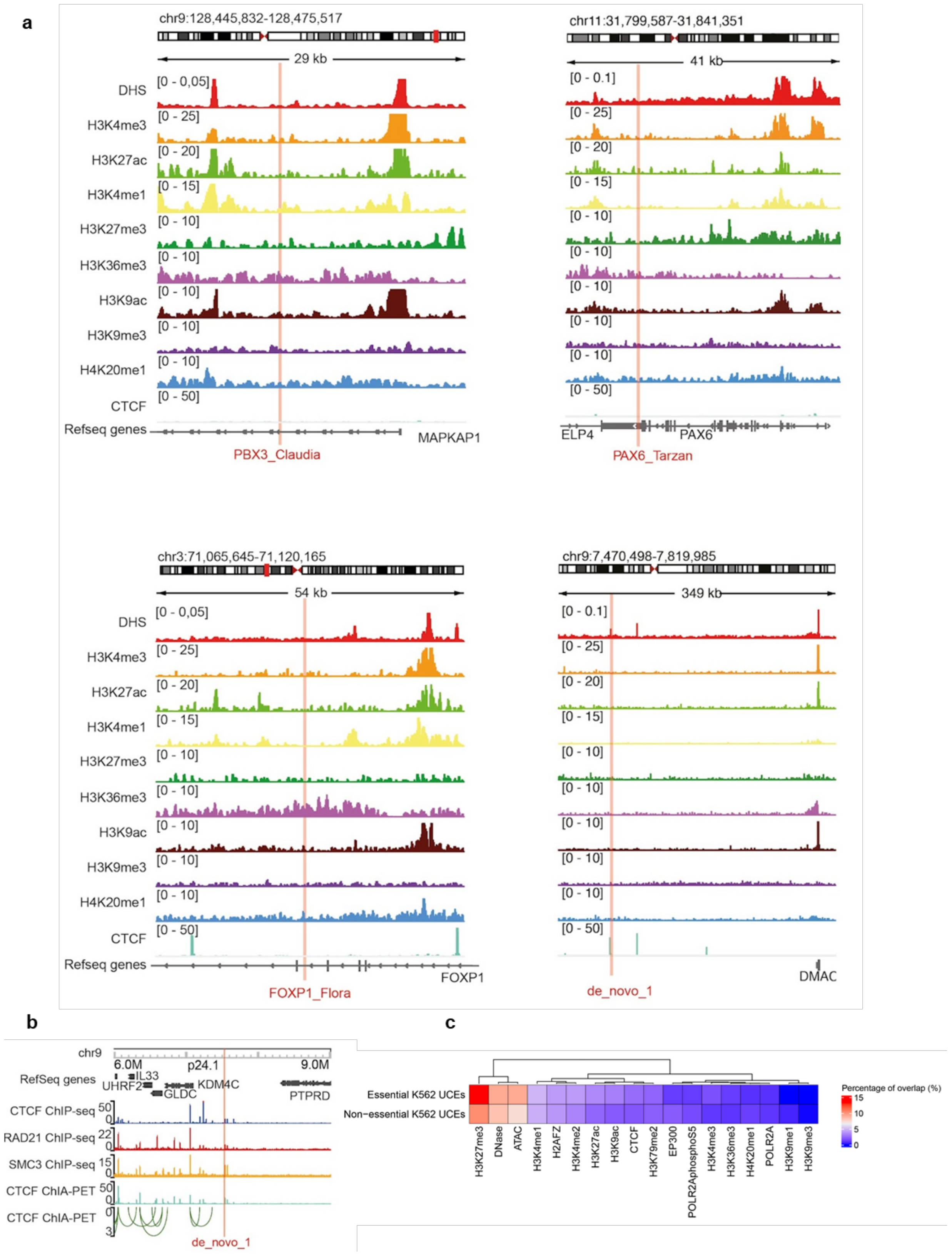

Supplementary Fig. 3 | Epigenetic patterns of validated essential K562 UCEs. a, Epigenetic signatures surrounding the UCEs PBX3_Cl, FOXP1_Fl, PAX6_Ta and the NCRE de_novo_1. **b,** Chromatin looping around NCRE de_novo_1, as shown by CTCF ChIA-PET loops. **c,** Epigenetic signatures of essential and non-essential UCEs in K562.

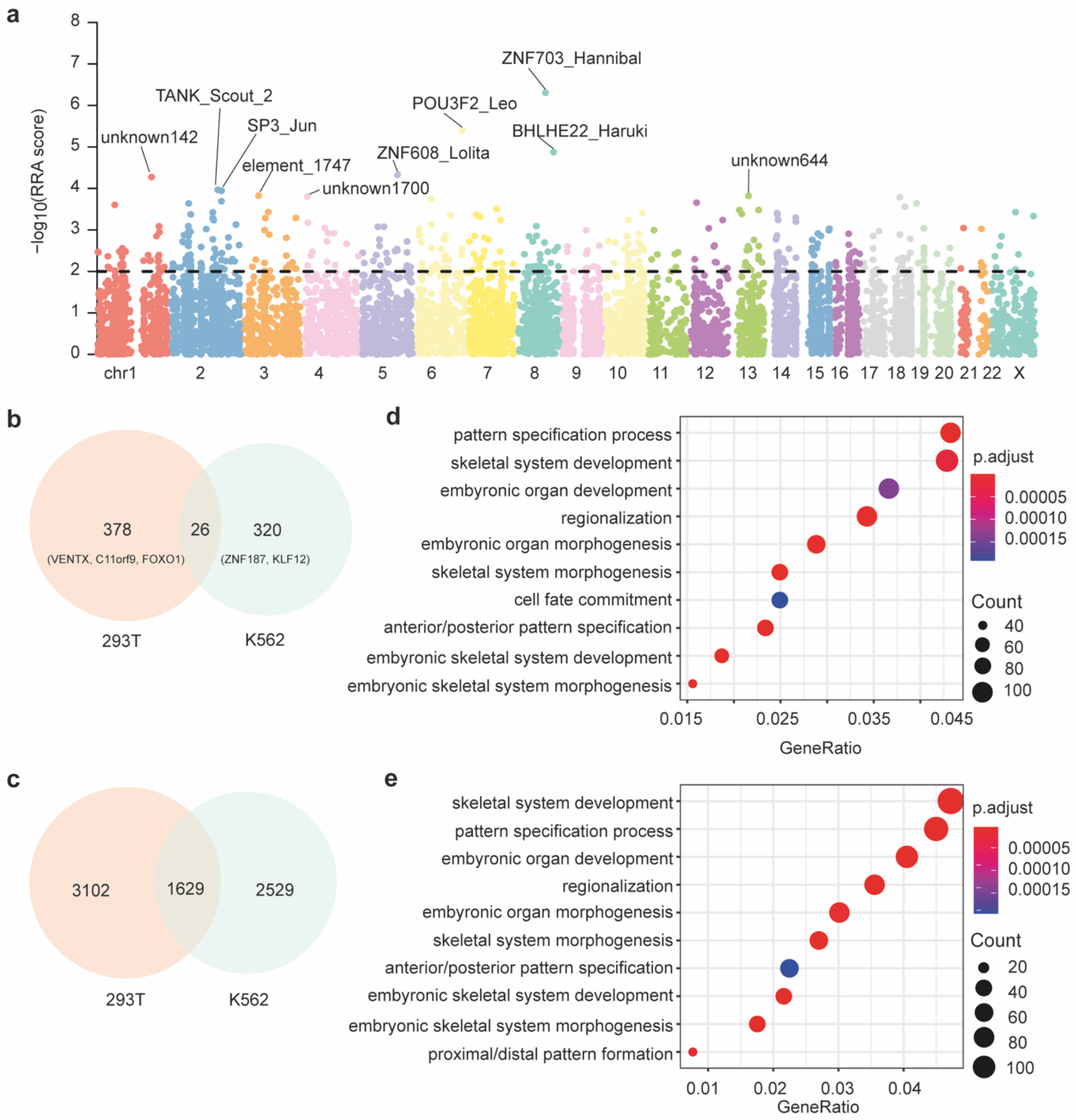

Supplementary Fig. 4 I Essential UCEs and NCREs shared between K562 and 293T cells. a, Essential UCEs and NCREs in 293T cells are identified using the dual-CRISPR screening. MAGeCK algorithm was used to identify significant hits depleted from the cells cultured for an additional 15 days compared to the initial population. The Manhattan plot was used to show the distribution of all the target regions. Significant hits were above the dashed line, indicating the cutoff MAGeCK RRA score of 0.01. Different colors represent different chromosomes. The top essential hits were shown. **b,** Venn diagram shows the shared essential UCEs and NCREs between K562 (346 UCEs and NCREs with RRA score < 0.01) and 293T (404 UCEs and NCREs with RRA score < 0.01) cell lines. Only 26 (<10%) of the essential UCEs and NCREs identified from the respective screenings were shared between K562 and 293T cells. TF motifs enriched in K562- and 293T-specific essential UCEs and NCREs were shown (P value < 10^{-5}). **c,** Venn diagram shows the genes around the essential UCEs and NCREs between K562 cells (4158 genes that are +/- 1Mbp around the essential UCEs and NCREs) and 293T (4731 genes that are +/- 1Mbp around the essential UCEs and NCREs) cells. 1629 (>30%) of the genes were shared between K562 and 293T cells. **d-e,** Over-representation analysis of Gene Ontology terms (Biological Process) based on the potential genes that might be regulated by the essential UCEs in 293T cells (**d**) and K562 cells (**e**).

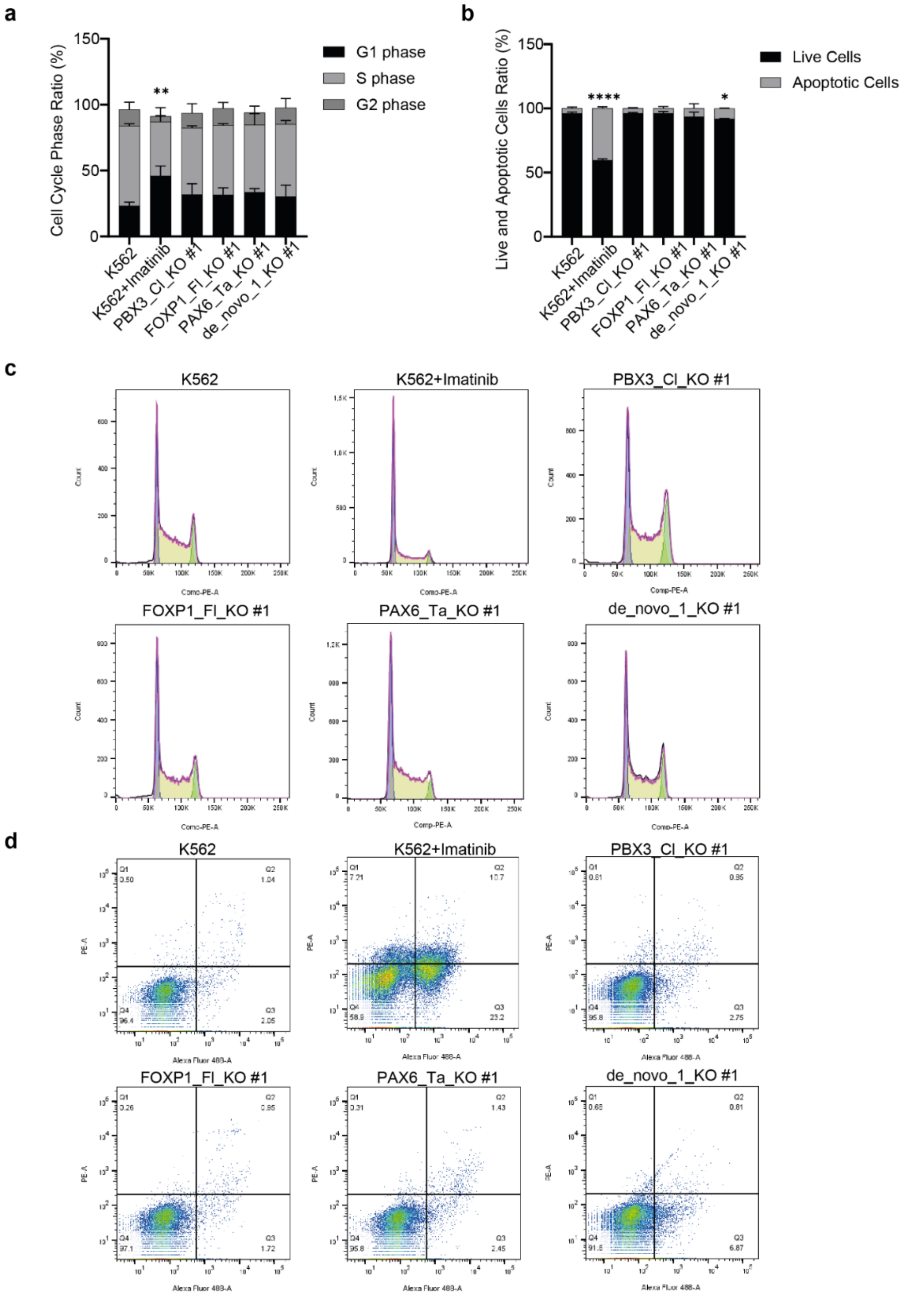

Supplementary Fig. 5 | Cell cycle and apoptosis analysis of essential UCE/NCRE-KO clones. a, Quantification of cell cycles in different UCE/NCRE-KO clones. Cells were fixed by ethanol and stained with PI. K562 cells treated with 10 µM imatinib for 1 day were arrested in the G1 phase and served as the positive control. (n = 3 biological independent samples; values are shown as the mean ± s.d.; **P < 0.01, calculated using one-way ANOVA). **b,** Quantification of live and apoptotic cells in different UCE/NCRE-KO clones. Cells were stained with PI and Annexin V-FITC (BioLegend 640914). K562 cells treated with 10 µM imatinib for 2 days went to apoptosis, and served as the positive control. PI-negative/Annexin V-positive and PI-positive/Annexin V-positive cells were considered apoptotic cells (n = 3 biological independent samples; values are shown as the mean ± s.d.; *P < 0.05, ****P < 0.0001, calculated using one-way ANOVA). **c,** FACS gating examples to quantify cell cycles. **d,** FACS gating examples to quantify live and apoptotic cells.

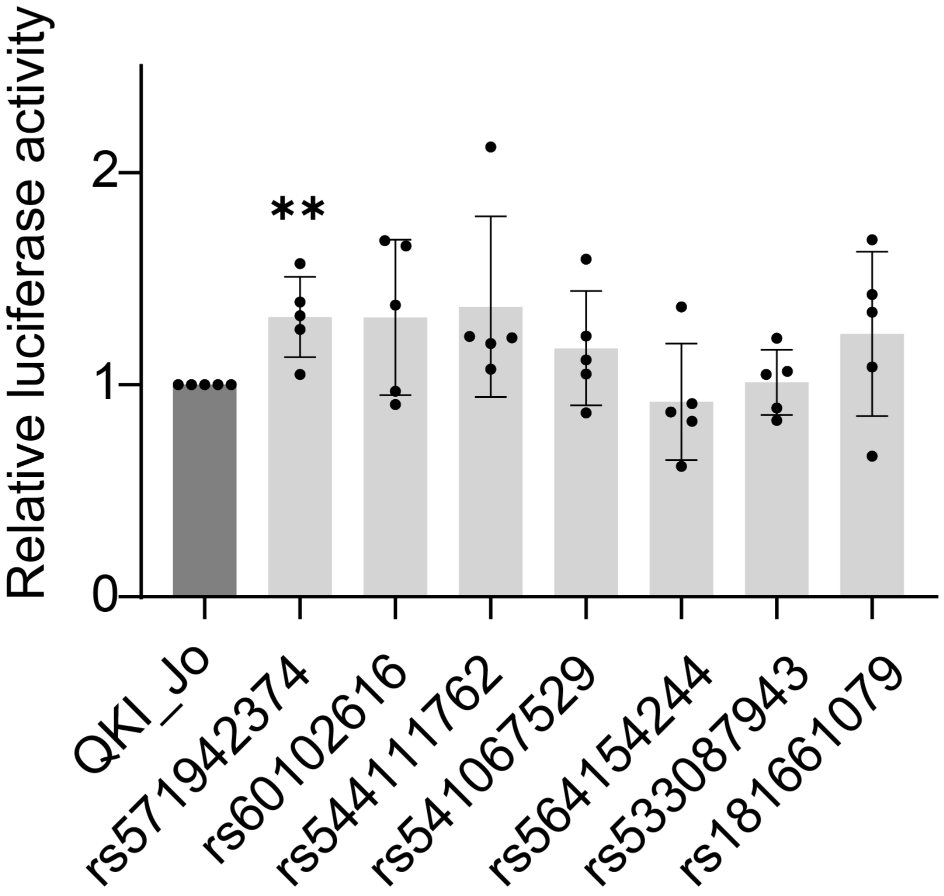

Supplementary Fig. 6 | Luciferase assays to determine the effects of SNPs on the silencer activity of QKI_Jo. The respective SNP was introduced to the QKI_Jo silencer region. The reference QKI_Jo silencer region was used as the control for the baseline luciferase activities. The y axis represents the relative unit of luciferase activity compared to that of the original QKI_Jo silencer in K562 cells ($n = 5$ biological independent samples; bars show mean value ± s.d.; rs571942374 $^{**}P = 0.0055$, rs60102616 $^{ns}P = 0.0893$, rs544111762 $^{ns}P = 0.0892$, rs541067529 $^{ns}P = 0.1919$, rs564154244 $^{ns}P = 0.5296$, rs533087943 $^{ns}P = 0.8771$, rs181661079 $^{ns}P = 0.2023$, calculated using two-tailed unpaired t-test).

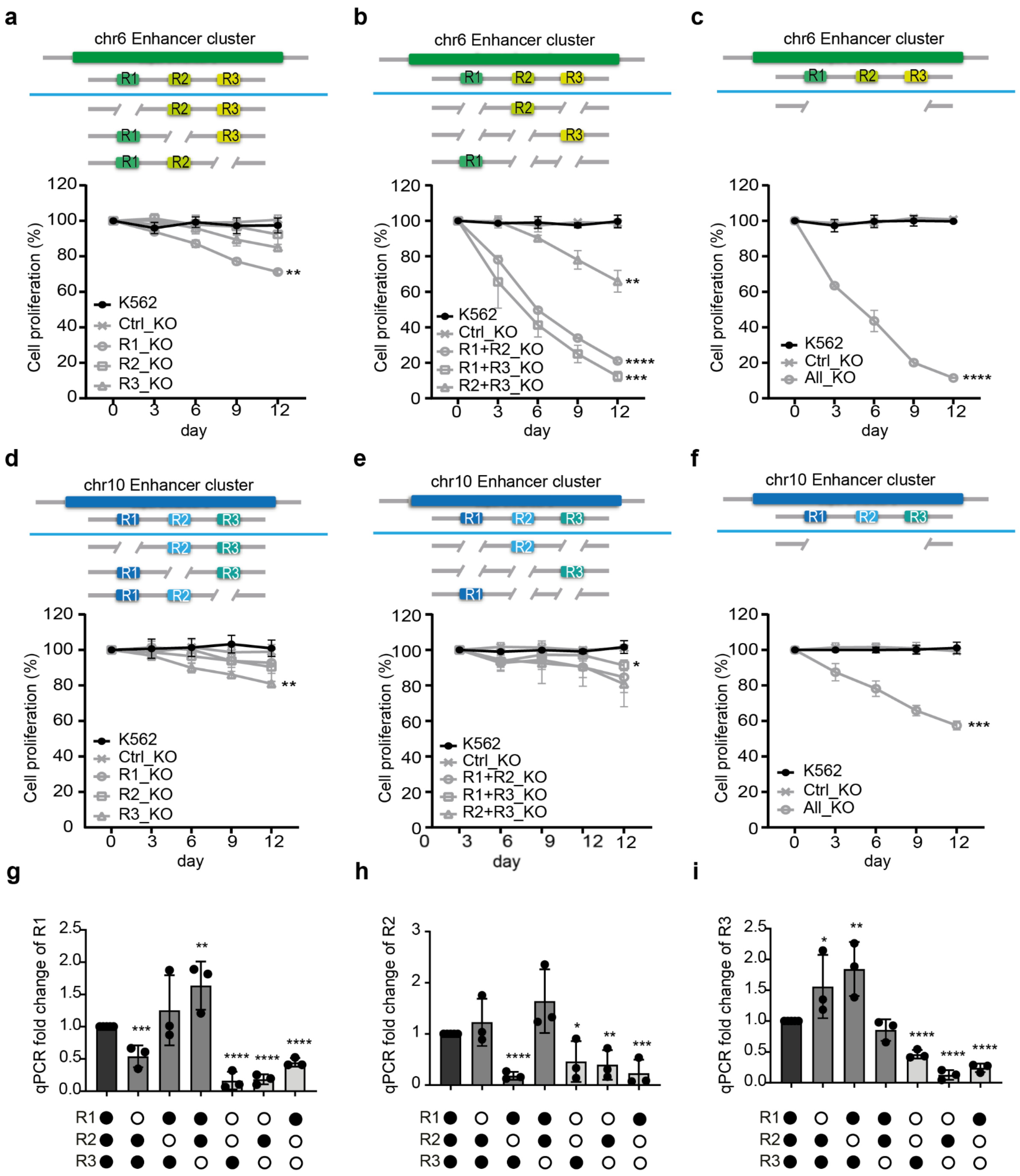

Supplementary Fig. 7 | Identifying essential enhancer clusters. a-c, Growth effect of one enhancer (**a**), two-enhancer (**b**), and three-enhancer (**c**) removal from the cluster in chromosome 6. Cell proliferation assay was performed by mixing the KO cell lines with cells expressing GFP at a 1:1 ratio. The changes in GFP percentage were monitored at indicated time points by FACS. Cells with dual-CRISPR guide RNAs targeting GFP sequences served as negative controls (Ctrl_KO). The y-axis represents the relative ratio of the GFP negative cells to the positive cells. The ratio of cells in the initial mixture was set as 100%. R1, R2 and R3 represent the three individual potential enhancers targeted by the dual-CRISPR libraries ($n = 3$ biological independent samples; values are shown as the mean ± s.d.; $^{**}P < 0.01$, $^{***}P < 0.001$, $^{****}P < 0.0001$, calculated using two-way ANOVA). **d-f,** Growth effect of one-enhancer (**d**), two-enhancer (**e**), and three-enhancer (**f**) removal from the cluster in chromosome 10. Cell proliferation assay was performed by mixing the KO cell lines with cells expressing GFP at a 1:1 ratio. The changes in GFP percentage were monitored at indicated time points by FACS. Cells with dual-CRISPR guide RNAs targeting GFP sequences served as negative controls (Ctrl_KO). The y-axis represents the relative ratio of the GFP negative cells to the positive cells. The ratio of cells in the initial mixture was set as 100%. R1, R2 and R3 represent the three individual potential enhancers targeted by the dual-CRISPR libraries ($n = 3$ biological independent samples; values are shown as the mean ± s.d.; $^{*}P < 0.05$, $^{**}P < 0.01$, $^{***}P < 0.001$, calculated using two-way ANOVA). **g-i,**

Transcription of eRNA from the individual enhancers (R1, R2 and R3) within the enhancer cluster on chromosome 22. Transcription of eRNAs of R1 (**g**), R2 (**h**) and R3 (**i**) in different enhancer KO clones were quantified by qPCR ($n = 3$ biological independent samples; values are shown as the mean value ± s.d. was shown for each bar; P values were calculated using unpaired t-test. *$P < 0.05$, **$P < 0.01$, *** $P < 0.001$, ****$P < 0.0001$, calculated using unpaired t-test).

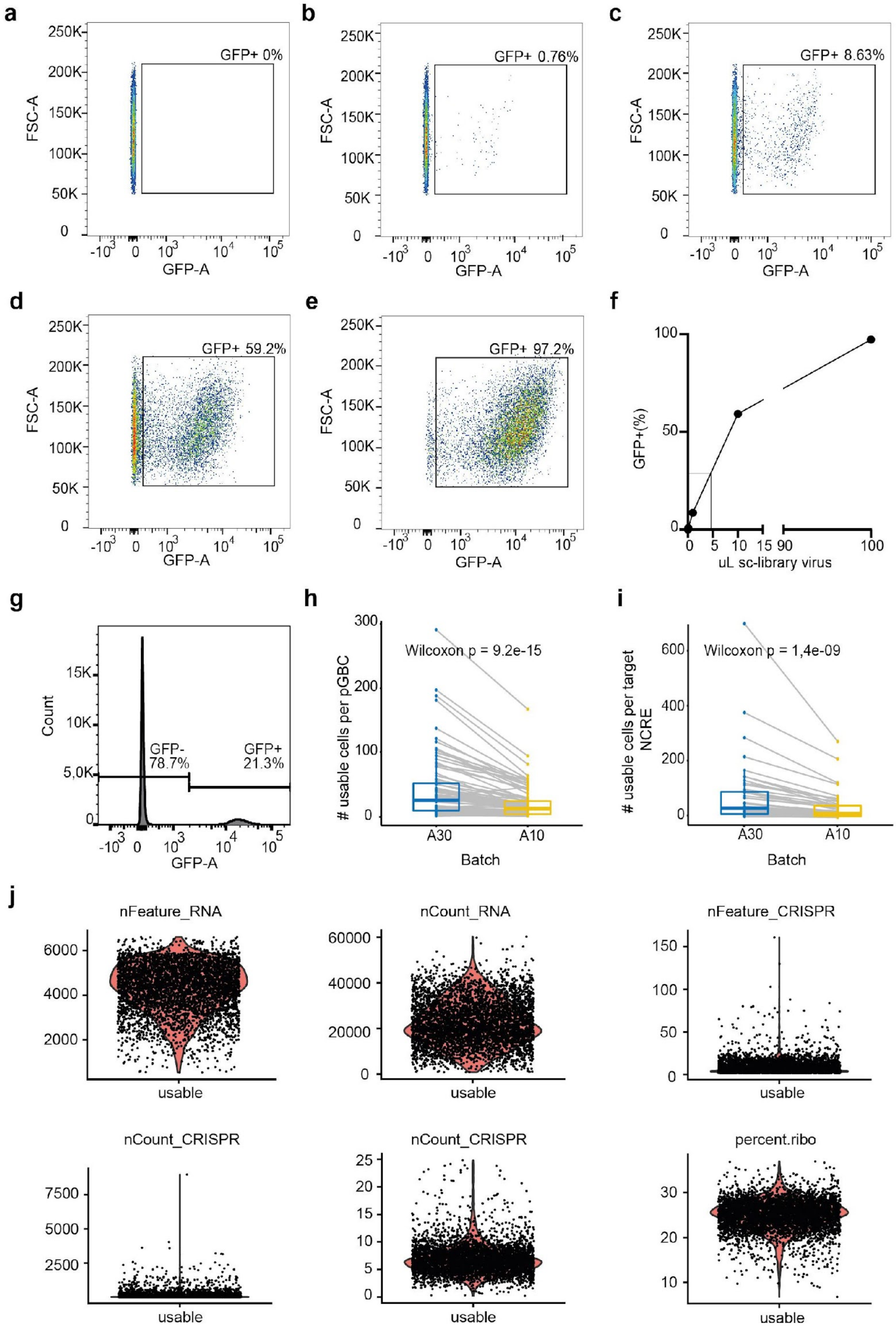

a
GFP+ 0%
FSC-A
GFP-A
b
GFP+ 0.76%
c
GFP+ 8.63%
d
GFP+ 59.2%
e
GFP+ 97.2%
f
GFP+(%)
uL sc-library virus
g
Count
GFP-
78.7%
GFP+
21.3%
h
Wilcoxon p = 9.2e-15
usable cells per pGBC
A30
A10
Batch
i
Wilcoxon p = 1,4e-09
usable cells per target NCRE
j
nFeature_RNA
nCount_RNA
nFeature_CRISPR
nCount_CRISPR
nCount_CRISPR
percent.ribo
usable

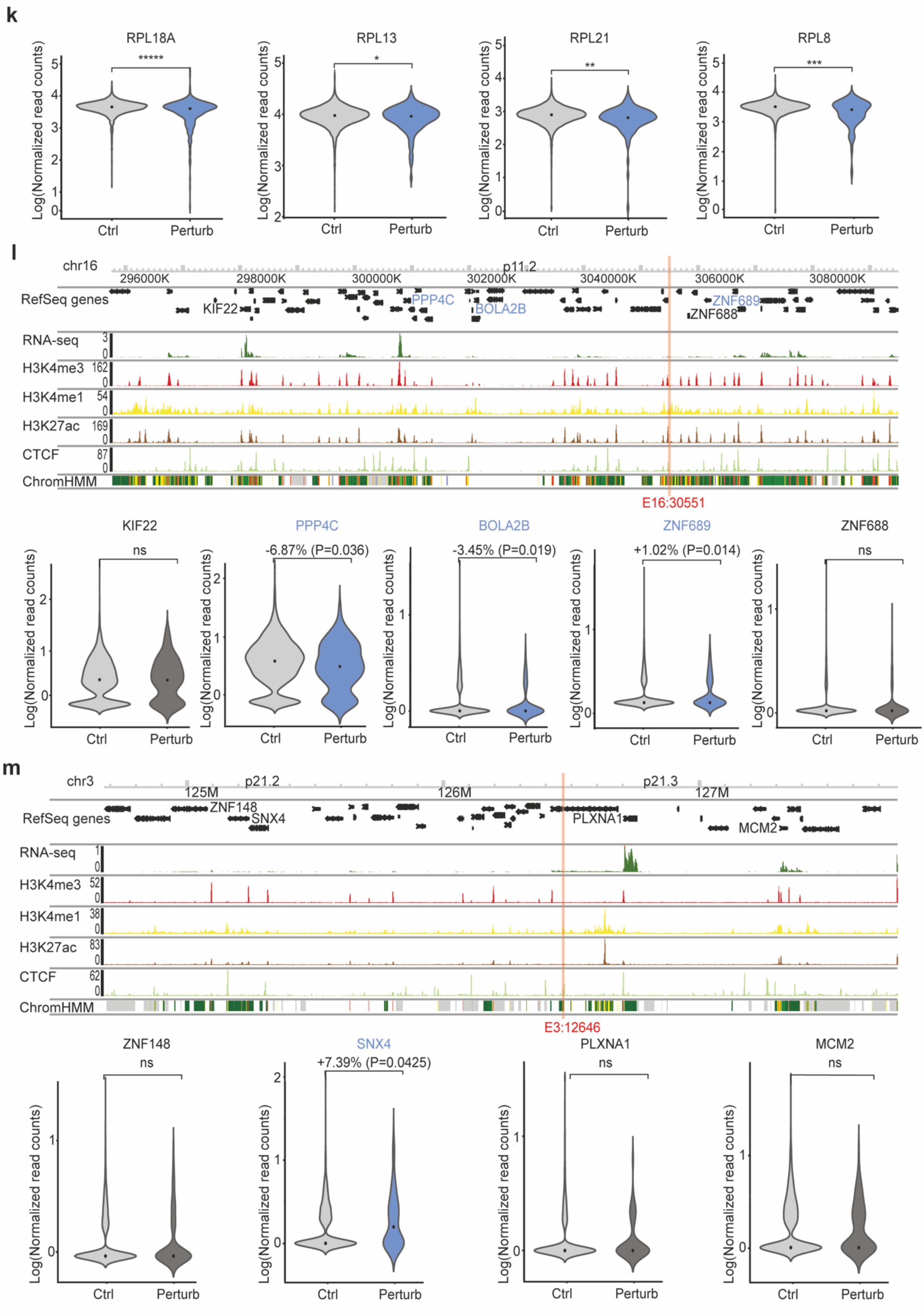
k
RPL18A
RPL13
RPL21
RPL8
*****
*
**
***
Log(Normalized read counts)
Ctrl
Perturb
l
chr16
p11.2
296000K
298000K
300000K
302000K
3040000K
306000K
3080000K
RefSeq genes
KIF22
PPP4C
BOLA2B
ZNF689
ZNF688
RNA-seq
H3K4me3
H3K4me1
H3K27ac
CTCF
ChromHMM
E16:30551
KIF22
PPP4C
BOLA2B
ZNF689
ZNF688
ns
-6.87% (P=0.036)
-3.45% (P=0.019)
+1.02% (P=0.014)
ns
m
chr3
p21.2
p21.3
125M
126M
127M
ZNF148
SNX4
PLXNA1
MCM2
E3:12646
ZNF148
SNX4
PLXNA1
MCM2
ns
+7.39% (P=0.0425)
ns
ns

n

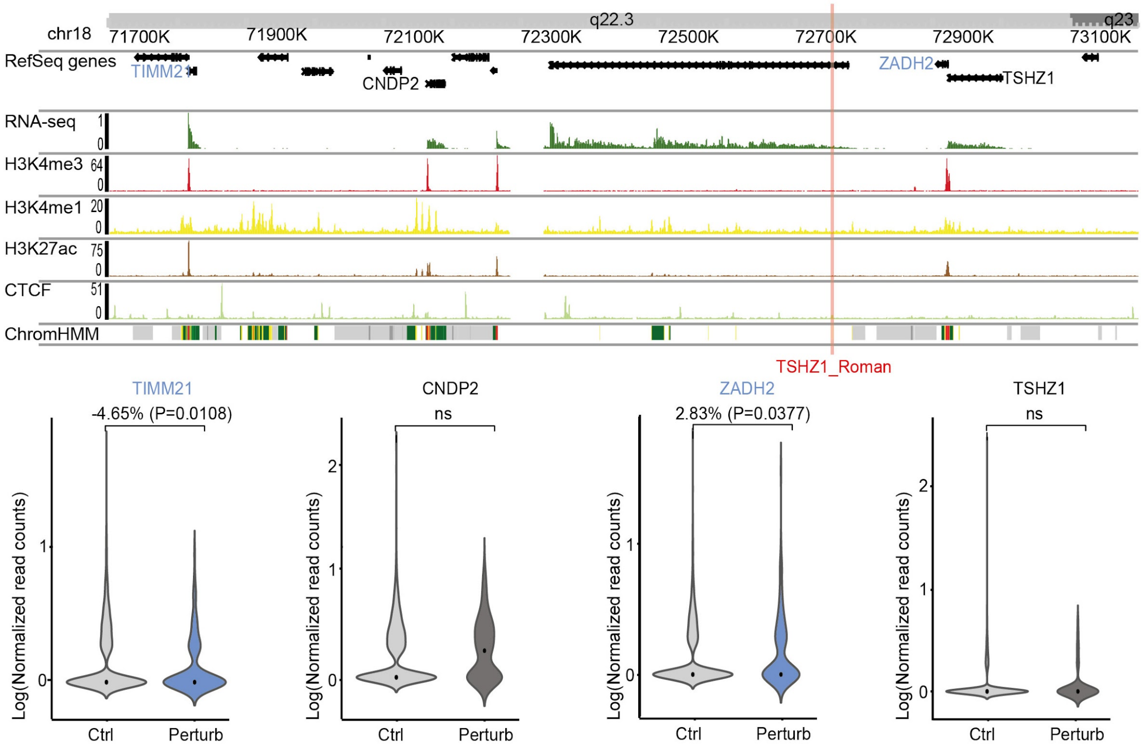

Supplementary Fig. 8 | Single-cell dual-CRISPR screening. a-f, Determining the lentiviral titer of the single-cell dual-CRISPR library (sc-library) in K562 cells, with a range of 0μL (**a**), 0.1μL (**b**), 1μL (**c**), 10μL (**d**) and 100μL (**e**) was tested. GFP expression from the sc-library was used as the marker to determine the virus infection rate. Individual plots show 0% GFP-expressing cells (**a**), 0,76% GFP-expressing cells (**b**), 8,63% GFP-expressing cells (**c**), 59,2% GFP-expressing cells (**d**), and 97,2% GFP-expressing cells (**e**), which was plotted in (**f**). A predicted MOI <0.3 using 5uL of sc-library lentivirus was then determined. **g,** Cells containing the sc-library for the downstream analysis. FACS showed that 20% of total cells had the sc-library, as monitored by GFP expression, representing an MOI of 0.2 in the final experiment. This ensured that every single cell contained only one pair of guide RNAs from the sc-library. **h,** Comparison of usable cells per paired guide RNA barcodes (pGBCs) between two batches of scRNA-seq data: A10 and A30 show 10,000 and 30,000 cells were loaded per 10X Genomics chip, respectively. 1,199 and 3,271 usable cells were retrieved from A10 and A30 batches, respectively. Wilcoxon signed-rank test was used to compare two datasets. (**i**) Comparison of usable cells per target NCRE (multiple pairs of guide RNA were included in the sc-library) between two batches of scRNA-seq data. Wilcoxon signed-rank test was used to compare two batch data. (**j**) Basic quality control of 4,470 single cells containing the sc-library after cell filtering and multiplets filtering. Violin plots show the total number of genes, total UMIs of transcripts, the total number of unique sgRNA, total UMIs of sgRNA detected, and the percentage of transcripts encoding mitochondrial and ribosomal genes detected in every single cell. **k,** The dual-CRISPRs targeting the promoters of ribosomal genes: RPL18A, RPL13, RPL21, and RPL8 served as the positive controls. The gene expression matrix was normalized using the LogNormalize method with a scale factor of 10,000. Identities of cell perturbation were assigned based on whether pGBCs were captured. Differential gene expression testing between perturbed cells (Perturb) and negative control cells (NCT) were done by the MAST algorithm with the number of gene UMIs observed per cell and the number of gRNA UMIs per cell as covariates (*****P<0.00001, ***P<0.001, **P<0.01, *P<0.05). **l-n,** The upper panel shows epigenetic signatures surrounding NCREs E16:30551 (**l**), E3:126465 (**m**), and TSHZ1_Roman (**n**), indicated by the red line. The lower panel shows the differential gene expression testing results using MAST. Violin plots show the normalized expression levels of candidate genes in perturbed and control cells ($P < 0.05$, calculated by MAST fitted model; ns: not significant).

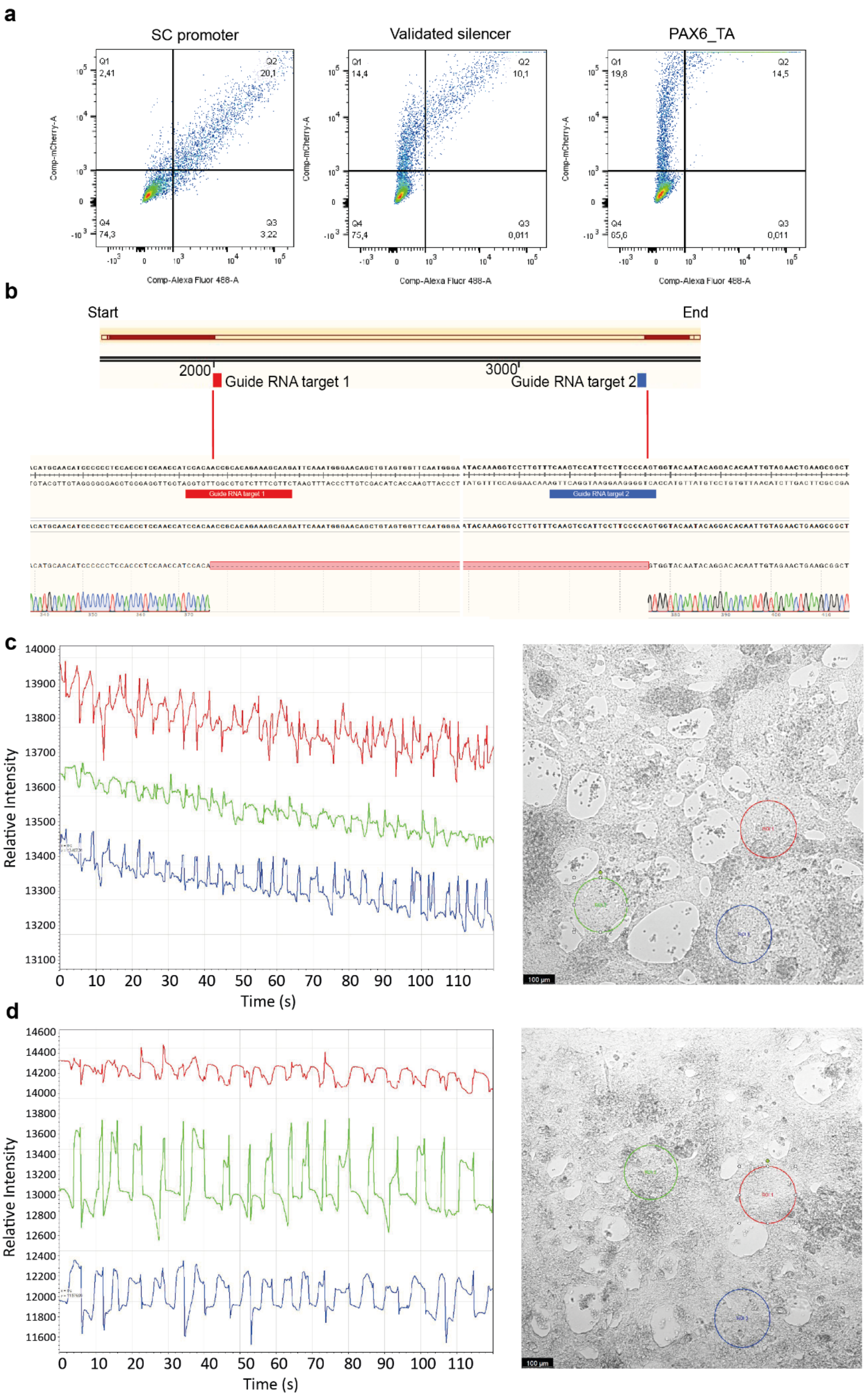

a
SC promoter
Validated silencer
PAX6_TA
Q1
Q2
Q3
Q4
2,41
20,1
3,22
74,3
14,4
10,1
0,011
75,4
19,8
14,5
0,011
65,6
Comp-mCherry-A
Comp-Alexa Fluor 488-A
b
Start
End
2000
3000
Guide RNA target 1
Guide RNA target 2
c
Relative Intensity
Time (s)
100 µm
d
Relative Intensity
Time (s)
100 µm

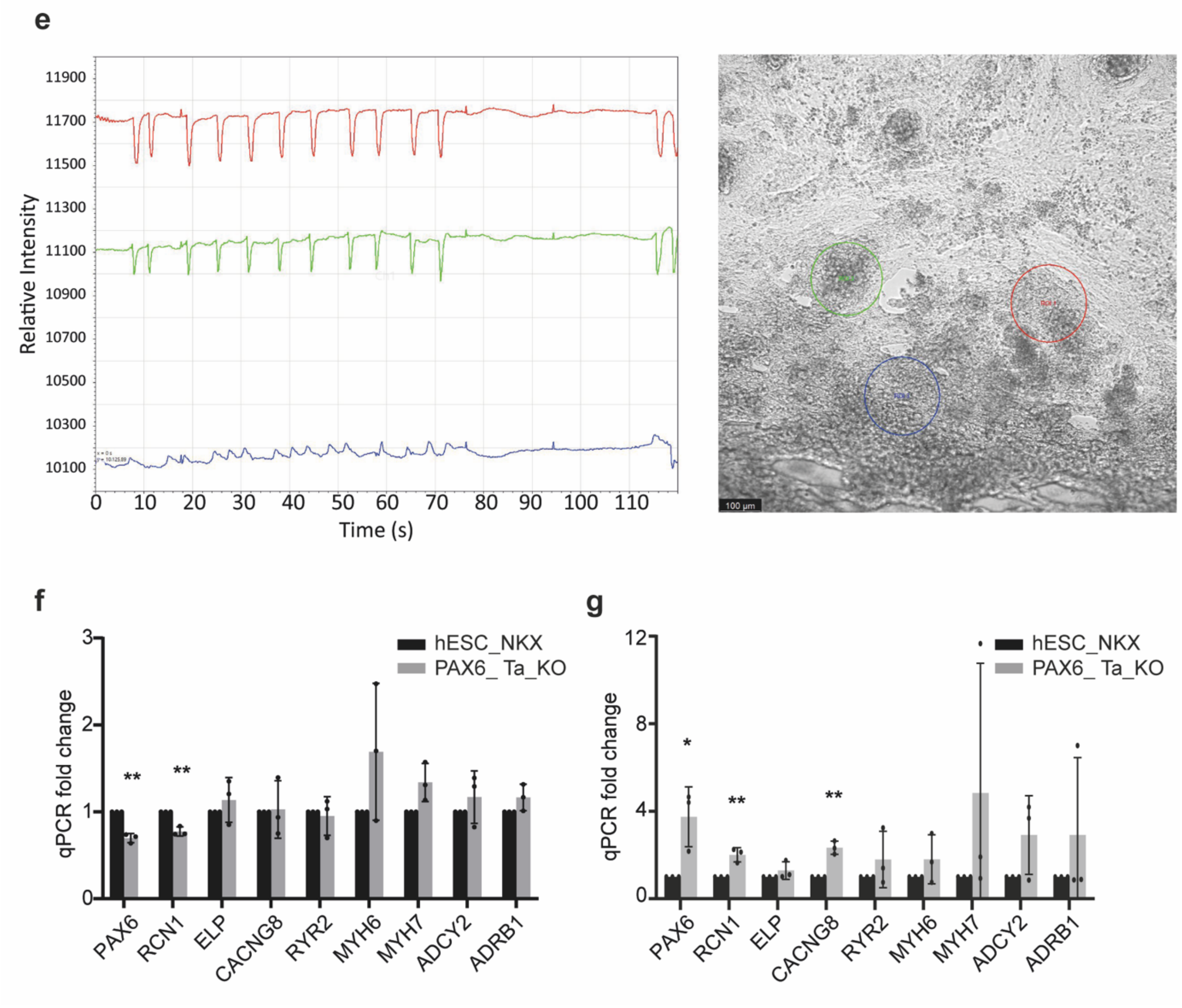

Supplementary Fig. 9 | Removing UCE PAX6 affecting cardiac development. a, FACS gating examples of the silencer reporter assay. **b,** Sanger sequencing of the PAX6_Ta region showed that the two guide RNAs made the deletion in NKX2-5eGFP/w hESC. **c-e,** The beating rhythm of cardiomyocyte cells 10 days post differentiation. NKX2-5eGFP/w hESC WT (**c**), AAVS1_KO (**d**) and PAX6_Ta_KO (**e**) were shown. Left panel: the *y*-axis represents the change of relative light intensity as a proxy for the beating rate within the region of interest (ROI). The *x*-axis represents the time frame. The right panel indicates the three ROIs selected for each condition. **f-g,** The transcription of heart-related genes PAX6, RCN1, ELP, CACNG8, RYR2, MYH6, MYH7, ADRY2, and ADRB1 was quantified by qPCR before (**f**) and after (**g**) differentiation in both NKX2-5eGFP/w hESC cells (hESC_NKX) and NKX2-5eGFP/w hESC PAX6_Ta KO bulk cells (PAX6_Ta_KO) ($n = 3$ biological independent samples, values are shown as the mean ± s.d. for each bar; $*P < 0.05$, $**P < 0.01$, calculated using unpaired t-test).

Additional Information

Source Data for Supplementary Fig. 1a | Unprocessed Western blot of Cas9-FLAG and Actin.

anti-FLAG

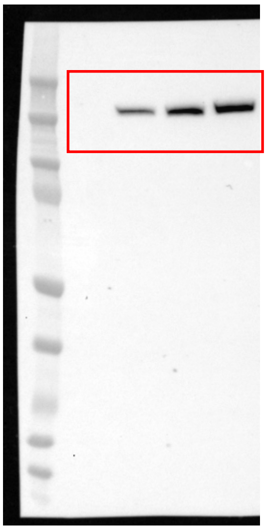

anti-actin

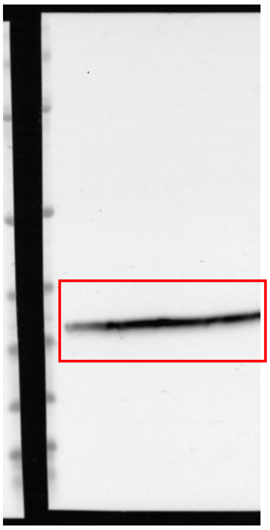

Supplement: Supplementary file 1 — Supplementary figures, tables and video captions, and unprocessed western blots. [file 41551_2024_1204_MOESM1_ESM.pdf]
