# Supplementary material for: Genome-wide Cas9-mediated screening of essential non-coding regulatory elements via libraries of paired single-guide RNAs
Source: Nat Biomed Eng. 2024 May 22;8(7):890–908. doi: 10.1038/s41551-024-01204-8 (PMC11310080; doi:10.1038/s41551-024-01204-8)
Supplement: Supplementary file 2 — Reporting Summary [file 41551_2024_1204_MOESM2_ESM.pdf]

nature portfolio

Corresponding author(s): Baoxu Pang

Last updated by author(s): Mar 13, 2024

Reporting Summary

Nature Portfolio wishes to improve the reproducibility of the work that we publish. This form provides structure for consistency and transparency in reporting. For further information on Nature Portfolio policies, see our Editorial Policies and the Editorial Policy Checklist.

Statistics

For all statistical analyses, confirm that the following items are present in the figure legend, table legend, main text, or Methods section.

n/a	Confirmed	
☐	☒	The exact sample size (*n*) for each experimental group/condition, given as a discrete number and unit of measurement
☐	☒	A statement on whether measurements were taken from distinct samples or whether the same sample was measured repeatedly
☐	☒	The statistical test(s) used AND whether they are one- or two-sided<br>*Only common tests should be described solely by name; describe more complex techniques in the Methods section.*
☐	☒	A description of all covariates tested
☐	☒	A description of any assumptions or corrections, such as tests of normality and adjustment for multiple comparisons
☐	☒	A full description of the statistical parameters including central tendency (e.g. means) or other basic estimates (e.g. regression coefficient) AND variation (e.g. standard deviation) or associated estimates of uncertainty (e.g. confidence intervals)
☐	☒	For null hypothesis testing, the test statistic (e.g. *F*, *t*, *r*) with confidence intervals, effect sizes, degrees of freedom and *P* value noted<br>*Give P values as exact values whenever suitable.*
☒	☐	For Bayesian analysis, information on the choice of priors and Markov chain Monte Carlo settings
☒	☐	For hierarchical and complex designs, identification of the appropriate level for tests and full reporting of outcomes
☐	☒	Estimates of effect sizes (e.g. Cohen's *d*, Pearson's *r*), indicating how they were calculated

Our web collection on statistics for biologists contains articles on many of the points above.

Software and code

Policy information about availability of computer code

Data collection

Sequencing was performed via the Illumina Hiseq4000 platform. Gels and blots were imaged by using the Bio-rad ChemiDoc Imaging System.

Data analysis

The R package CRISPRseek was used to search for potential protospacers sequences with PAM NGG pattern as the potential CRISPR targeting regions.

Cutadapt 3.4 was used to extract the unique 20nt protospacer sequences from each pair of guide RNA sequences by locating the U6/H1 promoter sequences from the 5' end and scaffold sequence from the 3' end of the 20nt protospacer sequence (U6 promoter, ATATATCTTGTGGAAAGGACGAAA; H1 promoter, ATAAGTTCTGTATGAGACCACTCTT). The trimmed reads were then mapped to the indexed paired protospacers references generated by Bowtie2 based on the library designs, and only aligned reads with mapping quality (MAPQ) score over 23 were used for downstream analyses.

MAGeCK RRA was used to identify the significant hits depleted after 15-day culture compared with the day 0 initial cell population, with the cutoff of RRA score < 0.01.

MAGeCK MLE was performed to identify NCREs that confer imatinib resistance, by comparing the 15-day imatinib-treated cell population, 15-day culture cell population and day 0 initial cell population. The NCREs whose loss confers Imatinib resistance were identified as regions that were positively selected (i.e., NCREs with beta scores > mean + 2×s.d.) in the 15-day imatinib-treated populations but are weakly selected in the 15-day culture populations.

The snakemake pipeline (https://github.com/snakemake-workflows/rna-seq-star-deseq2) was used to process the bulk RNA-seq sample. The gene pathway enrichment analyses based on GO term and KEGG pathway definitions were conducted by clusterProfiler with the cutoff of

FDR < 0.25.
3D Genome Browser (www.3dgenome.org) and WashU Epigenome Browser (https://epigenomegateway.wustl.edu/browser/) were used for data visualization.

Cell Ranger 6.0.1 pipeline was used to process Chromium single-cell data to align reads, generate feature-barcode matrices for the mRNAs, and capture guide RNAs. Seurat 4.0.2 was used to process the single-cell RNA-seq data.

The code for processing the pooled dual-CRISPR screen data is available on GitHub: https://github.com/PangLab/DualCRISPR_pooled_screen_snakemake_pipeline.

For manuscripts utilizing custom algorithms or software that are central to the research but not yet described in published literature, software must be made available to editors and reviewers. We strongly encourage code deposition in a community repository (e.g. GitHub). See the Nature Portfolio guidelines for submitting code & software for further information.

Data

Policy information about availability of data

All manuscripts must include a data availability statement. This statement should provide the following information, where applicable:

- Accession codes, unique identifiers, or web links for publicly available datasets
- A description of any restrictions on data availability
- For clinical datasets or third party data, please ensure that the statement adheres to our policy

Pooled screen and scRNA-seq sequencing data are available at GEO under accession code GSE254241. RNA-seq data are available at GEO under accession code GSE247234. The raw and analysed datasets generated during the study are available for research purposes from the corresponding authors on reasonable request. We plan to make the reagents widely available to the academic community through Addgene.

Research involving human participants, their data, or biological material

Policy information about studies with human participants or human data. See also policy information about sex, gender (identity/presentation), and sexual orientation and race, ethnicity and racism.

Reporting on sex and gender	The study did not involve human participants.
Reporting on race, ethnicity, or other socially relevant groupings	–
Population characteristics	–
Recruitment	–
Ethics oversight	–

Note that full information on the approval of the study protocol must also be provided in the manuscript.

Field-specific reporting

Please select the one below that is the best fit for your research. If you are not sure, read the appropriate sections before making your selection.

☒ Life sciences ☐ Behavioural & social sciences ☐ Ecological, evolutionary & environmental sciences

For a reference copy of the document with all sections, see nature.com/documents/nr-reporting-summary-flat.pdf

Life sciences study design

All studies must disclose on these points even when the disclosure is negative.

Sample size

For dual-CRISPR-sequencing experiments, K562/Cas9 cells were infected with the respective virus libraries at a multiplicity of infection 0.2 by spin-infection. For spin-infection, 3 million cells in each well of a 12-well plate were infected in 1 ml of medium containing 8 μg/ml of polybrene. In total, four plates were used for each infection to infect a total of 150 million cells, which would result in ~300× to ~500× coverage of the dual-CRISPR libraries.

For single-cell RNA-sequencing experiments, cells were loaded to recover a median coverage of 500 cells per guide. To prepare for single-cell processing, 500.000 cells at a concentration of 1,500 cells /ml in 0.04% BSA-PBS were used for single-cell RNA and guide RNA capture according to the 10x Genomics protocol.

Data exclusions

Pooled dual-CRISPR screen: puromycin selected cells were used for pooled dual CRISPR screen. Plasmid DNA did not contain the correct promoter and scaffold sequences ligations were not considered in pooled screen analysis.

Single-cell dual-CRISPR screen: GFP-expressing cell were sorted and loaded for direct gRNA capture and scRNA-seq assay. Cells with low UMI

	counts were removed from single cell CRISPR analysis in cellranger pipeline. Only single cells with captured correct guide-RNA pairs were kept for downstream analysis (4,470 cells).
Replication	All PCR assays, Western blots, luciferase assays and qPCR assays were performed at least 2–3 times using biological replicates performed at different days. Similar observation was made for each replicate.
Randomization	Randomization was not relevant to the study, as it was based on comparing defined CRISPR-Cas9 edited cells under distinct time points or treatment conditions.
Blinding	Blinding was not relevant to the study, as it was based on objective quantitative methods.

Reporting for specific materials, systems and methods

We require information from authors about some types of materials, experimental systems and methods used in many studies. Here, indicate whether each material, system or method listed is relevant to your study. If you are not sure if a list item applies to your research, read the appropriate section before selecting a response.

Materials & experimental systems

n/a	Involved in the study	
☐	☒	Antibodies
☐	☒	Eukaryotic cell lines
☒	☐	Palaeontology and archaeology
☒	☐	Animals and other organisms
☒	☐	Clinical data
☒	☐	Dual use research of concern
☒	☐	Plants

Methods

n/a	Involved in the study	
☒	☐	ChIP-seq
☐	☒	Flow cytometry
☒	☐	MRI-based neuroimaging

Antibodies

Antibodies used	Actin antibody # MA5-15452, Thermo Fisher Scientific (1:5000 dilution); Monoclonal ANTI-FLAG M2 antibody # F1804, Sigma (1:1000 dilution)
Validation	Actin antibody # MA5-15452<br>Species Reactivity: Hamster, Human, Mouse, Non-human primate, Rat<br>Specificity: MA5-15452 targets beta-Actin in FACS, IF, and WB applications and shows reactivity with Hamster, Human, mouse, Non-human primate, and Rat samples.The MA5-15452 immunogen is synthetic peptide corresponding to amino-terminal residues of human beta-Actin, conjugated to KLH.MA5-15452 detects beta-Actin which has a predicted molecular weight of approximately 42kDa.<br>Monoclonal ANTI-FLAG M2 antibody # F1804<br>Monoclonal ANTI-FLAG M2 antibody produced in mouse, 1 mg/mL, clone M2, affinity isolated antibody, buffered aqueous solution (50% glycerol, 10 mM sodium phosphate, and 150 mM NaCl, pH 7.4). Detects a single band of protein on a Western Blot from mammalian crude cell lysates.

Eukaryotic cell lines

Policy information about cell lines and Sex and Gender in Research

Cell line source(s)	K562 and 293T cells were all from the cell-line collections of the ENCODE project. hESC NKX2-5eGFP/w cells were from the authors of Nat Methods 8, 1037–1040 (2011).
Authentication	Cell lines from ENCODE were purchased from ATCC. ATCC performs routine cell-line authentication, and ENCODE maintains stocks of previously authenticated cell lines. hESC NKX2-5eGFP/w were authenticated by the providers.
Mycoplasma contamination	All cell lines tested negative for mycoplasma contamination.
Commonly misidentified lines<br>(See ICLAC register)	No commonly misidentified cells lines were used.

Flow Cytometry

Plots

Confirm that:

☒ The axis labels state the marker and fluorochrome used (e.g. CD4-FITC).

☒ The axis scales are clearly visible. Include numbers along axes only for bottom left plot of group (a 'group' is an analysis of identical markers).

☒ All plots are contour plots with outliers or pseudocolor plots.

☒ A numerical value for number of cells or percentage (with statistics) is provided.

Methodology

Sample preparation	K562 cells were analysed by flow cytometry or purified by fluorescence-activated cell sorting after lentiviral transduction and growth in standard culture conditions. GFP expression was used as a marker of successful transduction.
Instrument	Flow-cytometry data were collected using one LSR II flow cytometers, and cell sorting was performed on a FACSAria2 (BD Biosciences).
Software	Flow-cytometry data were collected using BD FACSDiva software (versions 8.0 and 8.0.1). FACSDiva was also used for cell sorting. And FlowJo_V10 was used for analysis.
Cell population abundance	–
Gating strategy	SSC/FSC gates were first applied to determine cells. Cells were then gated to separate GFP+ from GFP- cells.

☒ Tick this box to confirm that a figure exemplifying the gating strategy is provided in the Supplementary Information.
