# Supplementary material for: Genome-wide Cas9-mediated screening of essential non-coding regulatory elements via libraries of paired single-guide RNAs
Source: Nat Biomed Eng. 2024 May 22;8(7):890–908. doi: 10.1038/s41551-024-01204-8 (PMC11310080; doi:10.1038/s41551-024-01204-8)

Fig.1b unprocessed gel image

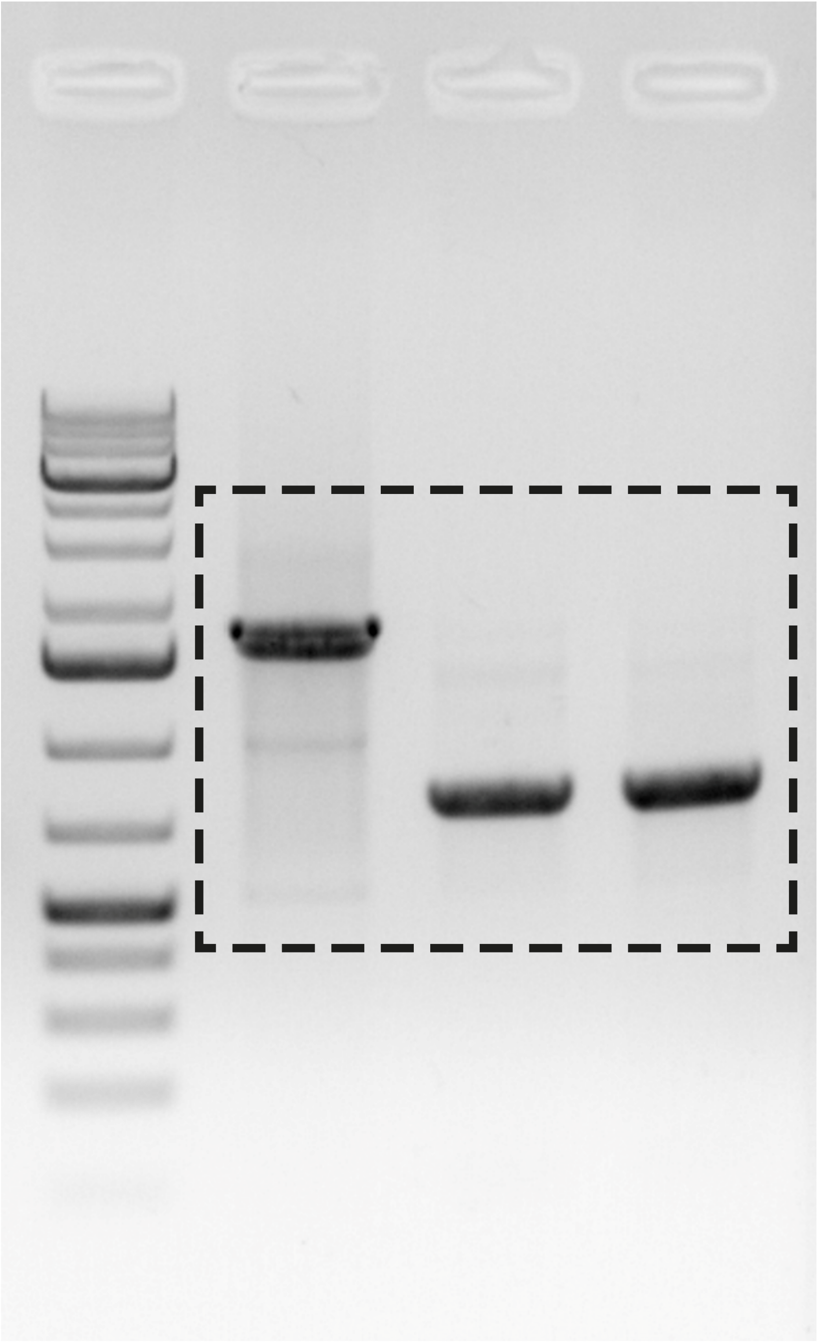

Fig.1c unprocessed gel image

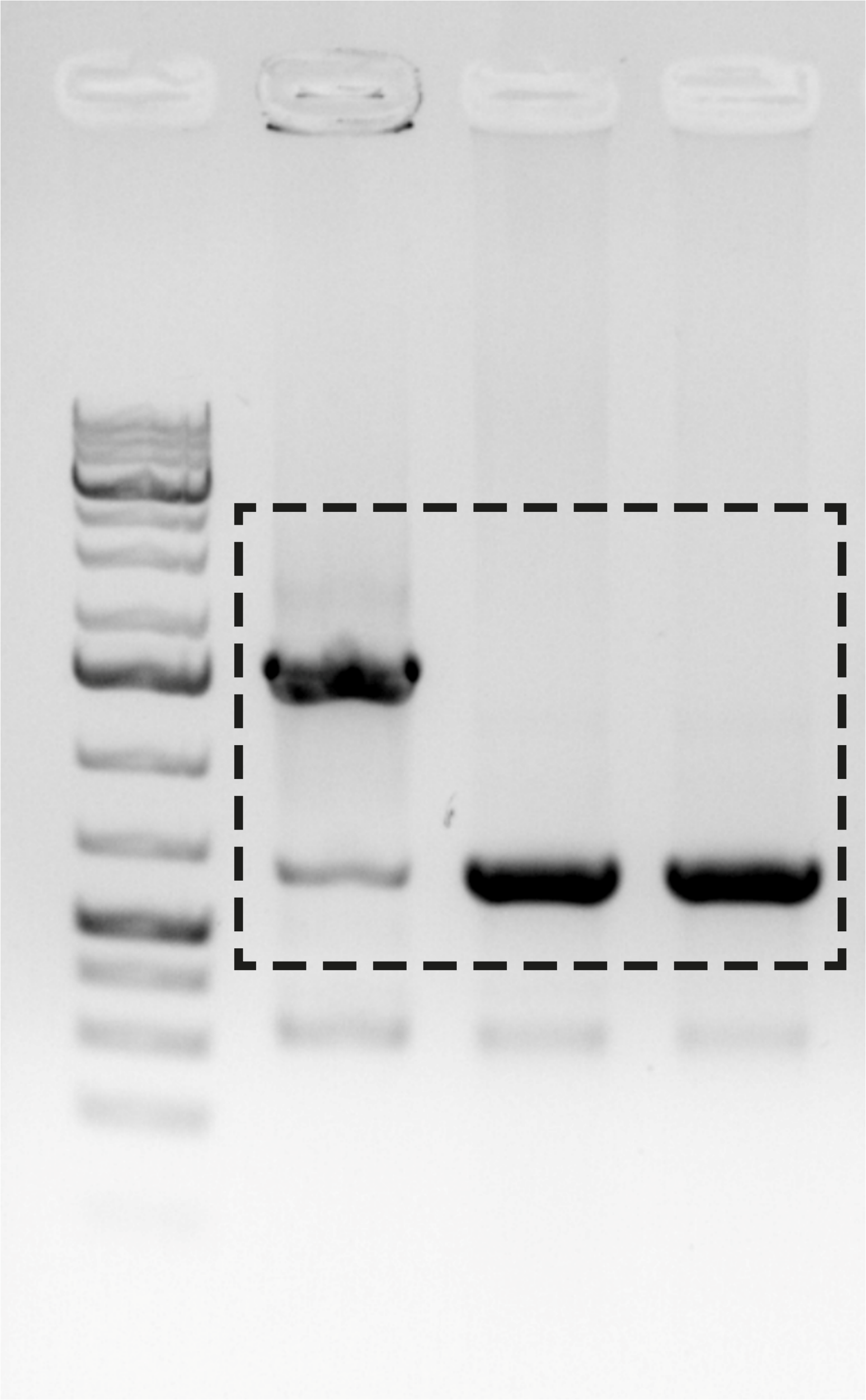

Fig.1d unprocessed gel image

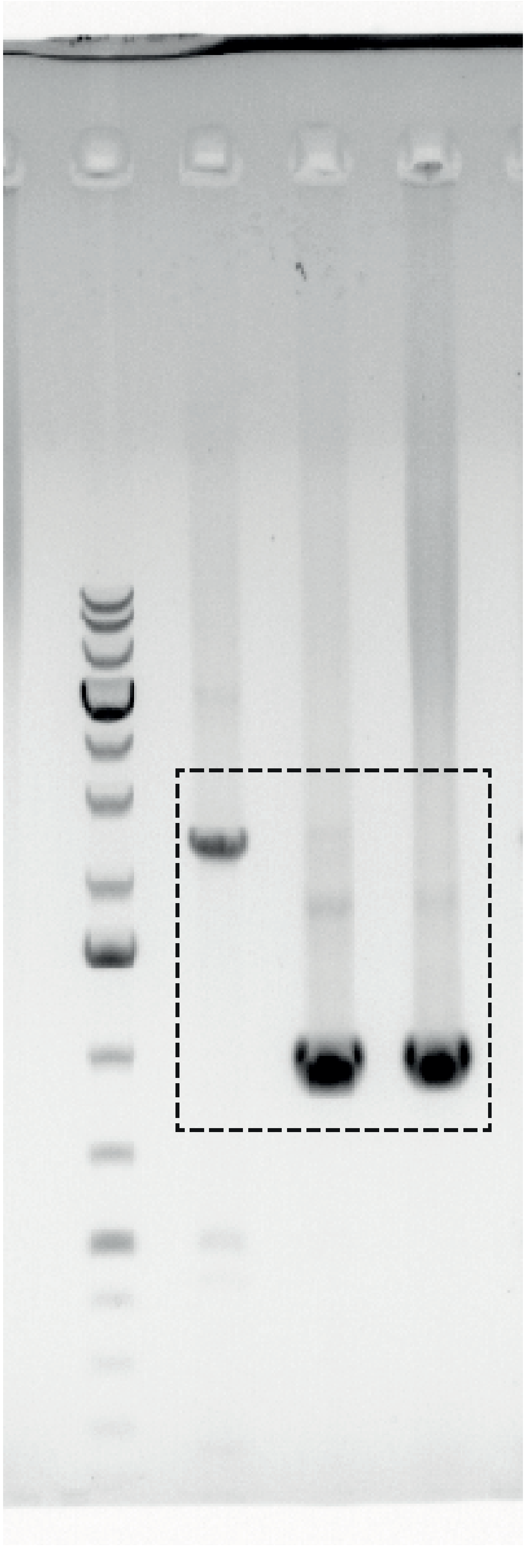

Fig.1e unprocessed gel image

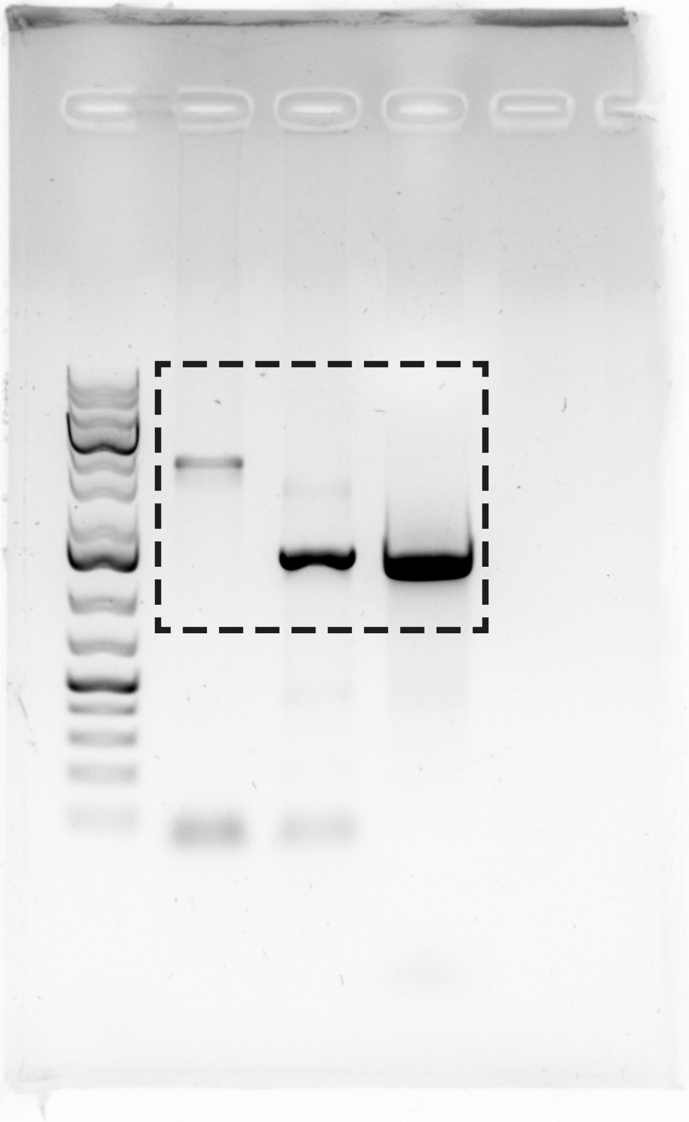

Supplement: Supplementary file 11 — Unprocessed gels. [file 41551_2024_1204_MOESM11_ESM.pdf]
