# Supplementary figures and images for: Genome-wide Cas9-mediated screening of essential non-coding regulatory elements via libraries of paired single-guide RNAs
Source: Nat Biomed Eng. 2024 May 22;8(7):890–908. doi: 10.1038/s41551-024-01204-8 (PMC11310080; doi:10.1038/s41551-024-01204-8)

Fig.2b unprocessed gel image

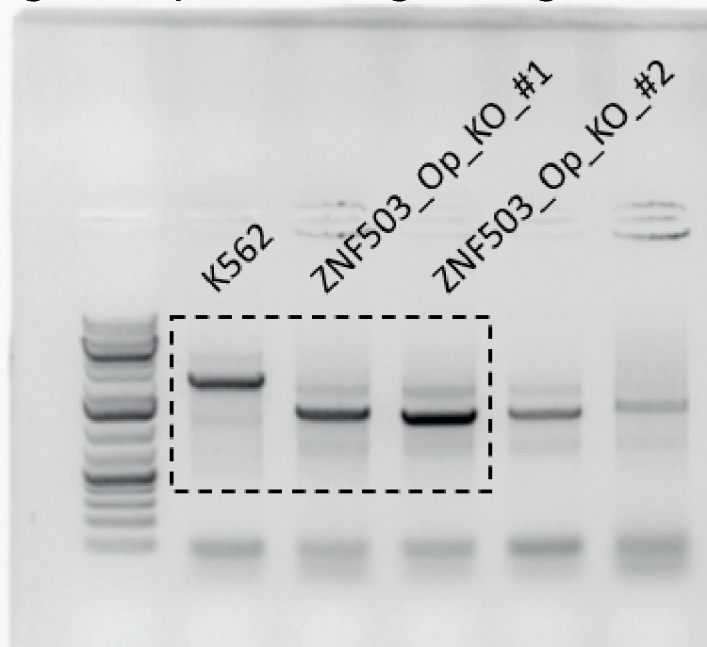

Fig,2c unprocessed gel image

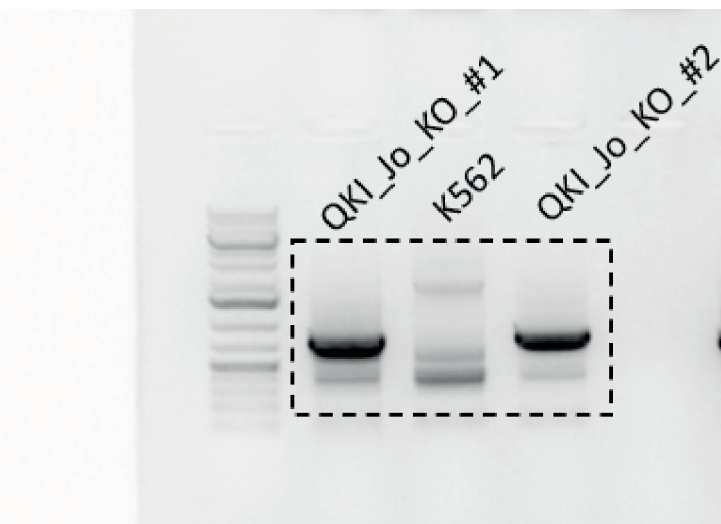

Supplement: Supplementary file 12 — Unprocessed gels. [file 41551_2024_1204_MOESM12_ESM.pdf]

Fig.6c unprocessed gel

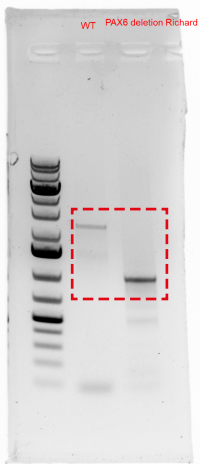

Supplement: Supplementary file 13 — Unprocessed gels. [file 41551_2024_1204_MOESM13_ESM.pdf]

Extended Data Fig.1b

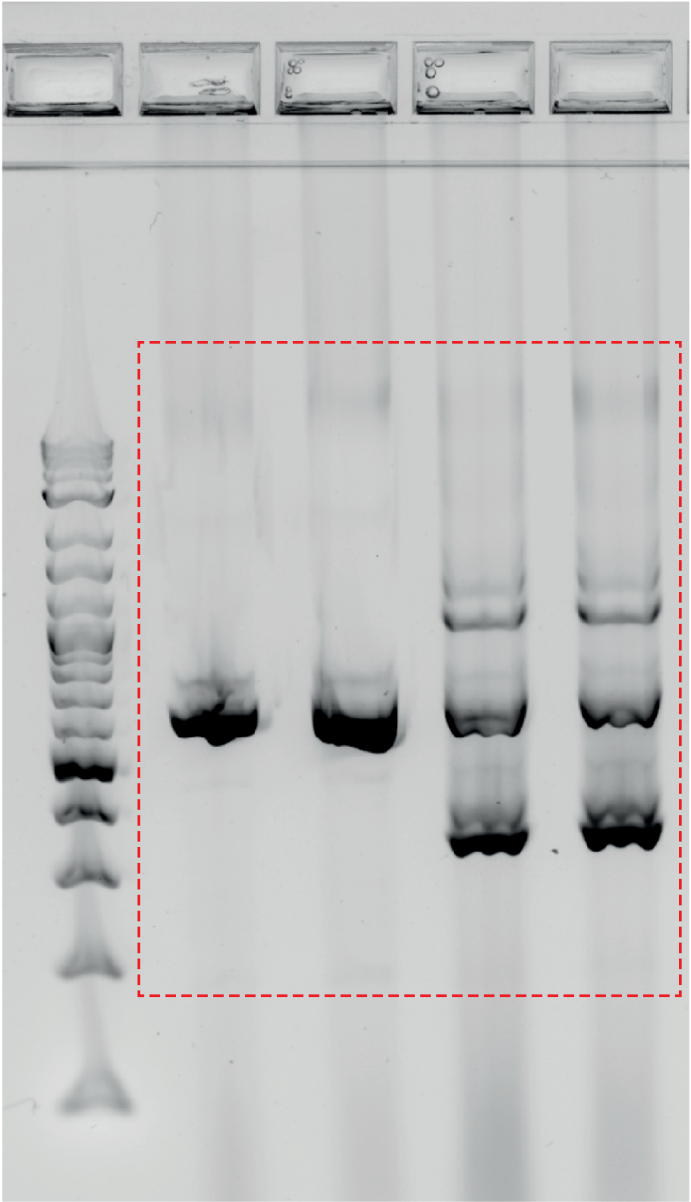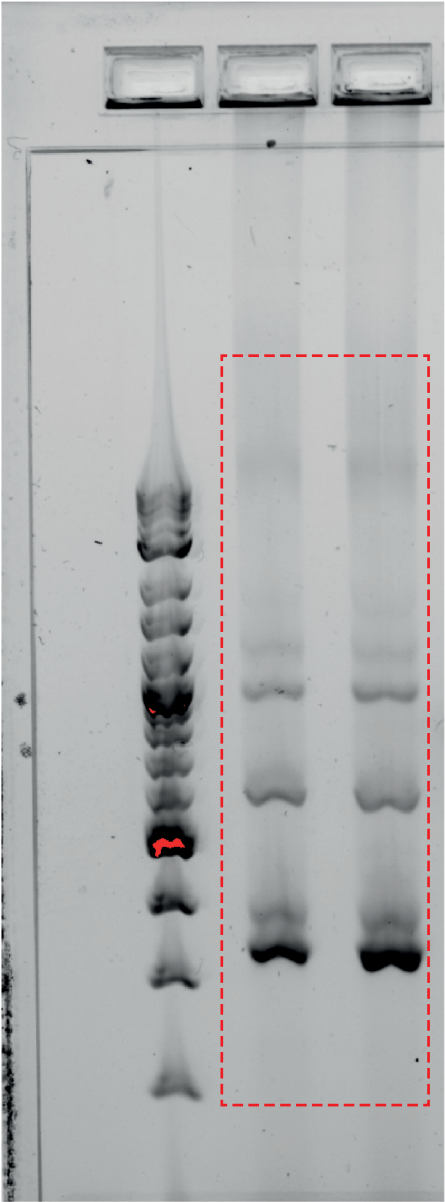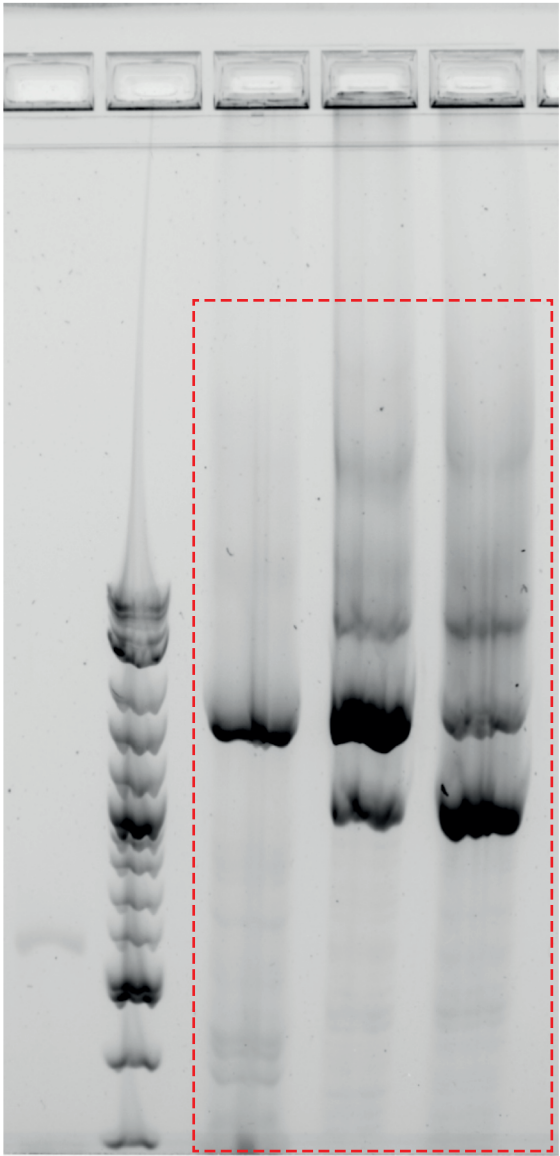

Supplement: Supplementary file 14 — Unprocessed gels. [file 41551_2024_1204_MOESM14_ESM.pdf]
